# Osteo-Arthritis

**Published:** 1907-12-14

**Authors:** 


### OSTEO-ARTHRITIS.

Clinically three main varieties of the disease are recognised. That most commonly seen in the out-patient departments of hospitals and of most interest to the surgeon is the chronic monarticular variety. It affects the larger articulations of the limbs, especially the hips and knees, and, unlike the polyarticular forms to be presently described, appears to attack males with greater frequency than females. The onset is characterised by pain of an insidious nature; it is first noticed in the morning or after the joint has been rested, but appears to work off after the joint has been used. Later, however, the pain becomes a constant phenomenon, becoming exacerbated at these times. Observant patients will also complain that when the joint is flexed they are conscious of a creaking sensation. If the joint is examined early in the disease little or no swelling may be found, but in the later stages the condition is always associated with well-marked deformity. This is due to the osteophytic outgrowths which occupy the periphery of the articulation; they may become so large that the anatomical outlines of the joint are completely lost. In addition to this, there is often some effusion into the synovial cavity, the fluid being secreted by the diseased synovial membrane. Curiously enough, some patients declare that the pain in the joint gets better rather than worse when effusion takes place.

The outlook is particularly unsatisfactory; the pain gets slowly but surely worse, and may finally keep the patient awake at night as well as prevent him following his occupation by day. Moreover the increase in the size of the osteophytes tends gradually to make the limitation of movement more and more pronounced until at last ankylosis occurs. This is hardly ever, except in the case of osteo-arthritis of the spine, a true bony ankylosis, but is rather a complete immobilisation of the joint owing to the interlocking of the osteophytes. As soon as this occurs the pain is diminished, and in some cases disappears almost entirely. This is easily intelligible, since the pain is definitely associated with movement in the degenerated joint. In fact, this state of affairs may be looked upon as a form of natural cure, as far as the patient's symptoms are concerned. It is true that the joint may not be fixed in the best possible position; but many patients regard this disadvantage with complacency when they consider the relief which it has afforded them. As in most diseases of joints, there is much wasting of the muscles on either side of the articulation. It might be thought that since the pathological lesions of osteo-arthritis are so characteristic the diagnosis would be easy, but there are several conditions which may simulate it. One is the arthropathy described by Virchow under the name of chondro-arthritis. It is apparently of syphilitic origin, and occurs as a late secondary or early tertiary phenomenon. As in osteo-arthritis, there is proliferation of cartilage cells on the articular surface. But these become softened and eroded instead of being calcified and worn away along the lines of movement in the joint. The eroded areas are rounded and punched out, and do not necessarily correspond to the sites of intra-articular pressure. Besides these points of difference there is neither pain nor crepitus on movement, and osteophytes are absent from the edges. Osteo-arthritis of the hip may be mistaken for intra-capsular fracture of the neck of the femur, especially if this is an impacted one. In both there is pain and crepitus on movement, and in both the top of the trochanter may be above Nélaton's line, in fracture because of the shortening of the limb, in osteo-arthritis on account of the osteophytic thickening of the great trochanter. But in the case of a fracture there is a history of some recent injury, however slight, with subsequent inability to walk. Careful measurement will demonstrate real shortening of the affected limb, and there is slackening of the ilio-tibial band. So there is really no excuse for confusing the two conditions.

The chronic polyarticular variety is more common in middle-aged women, and affects the joints of the hands. In each affected joint the typical signs described above are found, and the phalanges themselves become stiff, swollen, and tender, and present small nodules of bony outgrowth which are known as Heberden's nodes. In additon, the whole of the fingers distal to the metacarpo-phalangeal articulations are adducted to the ulnar side. The skin appears to undergo trophic changes and becomes smooth and shiny over the joints, while the palmar surface is cold and clammy. The acute polyarticular variety attacks the same joints as the last, but occurs generally in young women, often after an acute fever. The joints affected are swollen, hot, and painful. Ulnar deflection of the hand is not a feature of the acute disease. The patient is nearly always anæmic, and often has an irregular pyrexia. Trophic changes in the skin are well marked. The outlook in these acute cases is not at all promising. The patient should, if possible, avoid all worry. She should be fed up, and the affected joints should be kept warm, but in spite of all one's care and attention this condition usually lapses into the chronic variety.

The treatment of chronic osteo-arthritis is at present peculiarly unsatisfactory. The only drugs which seem to have any effect on the disease are potassium iodide and guaiacum. These may be combined in one prescription, but it seems that their effect is analgesic rather than curative. Experiments have lately been made in the treatment of these cases by Bier's method of passive congestion. An elastic ligature is applied round the limb proximal to the affected joint. The skin is protected by previously wrapping round it some wool or lint. The elastic bandage is applied just so tightly as to constrict the venous, but not the arterial circulation. Some patients can stand the continuous application of the bandage, but experiment seems to show that more benefit is derived if the bandage is applied at regular intervals for short periods. This form of treatment has not yet been given a thorough trial. All that can be said at present is that in some cases there is a very definite improvement, and that in the great majority the pain is relieved. Of the known forms of treatment perhaps the best is to send the patient to Bath to bathe in the naturally-heated water, and have hot under-current douches applied to the affected joint.

